# Exposure to an enriched environment modulates the synaptic vesicle cycle in a mouse spinal cord injury model

**DOI:** 10.1038/s41598-024-62112-0

**Published:** 2024-05-25

**Authors:** Jeehyun Yoo, Ji Cheol Shin, Kil-Byung Lim, Se Hoon Kim, Hyun Seok Kim, Sung Hoon Kim, Dawoon Baek, Seongmoon Jo, Jinyoung Kim, Ahreum Baek, Sung-Rae Cho

**Affiliations:** 1 Department of Rehabilitation Medicine, Ilsan Paik Hospital, Inje University, Gyeonggi-do, South Korea; 2 Department of Medicine, Yonsei University College of Medicine, Seoul, South Korea; 3 Department and Research Institute of Rehabilitation Medicine, Yonsei University College of Medicine, Seoul, South Korea; 4 Department of Pathology, Yonsei University College of Medicine, Seoul, South Korea; 5 Department of Biomedical Sciences, Yonsei University College of Medicine, Seoul, South Korea; 6 Department of Rehabilitation Medicine, Yonsei University Wonju College of Medicine, Wonju, South Korea; 7 Graduate School of Medical Science, Brain Korea 21 Project, Yonsei University College of Medicine, Seoul, South Korea; 8 Graduate Program of Biomedical Engineering, Yonsei University College of Medicine, Seoul, South Korea; 9 Rehabilitation Institute of Neuromuscular Disease, Yonsei University College of Medicine, Seoul, South Korea

**Keywords:** Enriched environment, Standard cage, Spinal cord injury, Synaptic vesicle cycle pathway, Rehabilitation training, Diseases, Neurology

## Abstract

Spinal cord injury (SCI) leads to motor and sensory impairment below the site of injury, thereby necessitating rehabilitation. An enriched environment (EE) increases social interaction and locomotor activity in a mouse model, similar to human rehabilitation. However, the impact of EE on presynaptic plasticity in gene expression levels remains unclear. Hence, this study aimed to investigate the therapeutic potential of EE in an SCI mouse model. Mice with spinal cord contusion were divided into two groups: those housed in standard cages (control) and those in EE conditions (EE). Each group was housed separately for either 2- or 8-weeks post-injury, after which RNA sequencing was performed and compared to a sham group (receiving only a dorsal laminectomy). The synaptic vesicle cycle (SVC) pathway and related genes showed significant downregulation after SCI at both time points. Subsequently, we investigated whether exposure to EE for 2- and 8-weeks post-SCI could modulate the SVC pathway and its related genes. Notably, exposure to EE for 8 weeks resulted in a marked reversal effect of SVC-related gene expression, along with stimulation of axon regeneration and mitigation of locomotor activity loss. Thus, prolonged exposure to EE increased presynaptic activity, fostering axon regeneration and functional improvement by modulating the SVC in the SCI mouse model. These findings suggest that EE exposure proves effective in inducing activity-dependent plasticity, offering a promising therapeutic approach akin to rehabilitation training in patients with SCI.

## Introduction

Following spinal cord injury (SCI), patients commonly experience motor and sensory impairments below the site of injury, and rehabilitation plays a crucial role in facilitating their functional recovery. Hebbian plasticity, characterized by activity-dependent changes that reinforce active circuits and weaken inactive ones in the central nervous system^1^, contributes to the functional recovery observed in patients with SCI undergoing rehabilitative training^2–4^.

An enriched environment (EE) typically consists of multiple mice housed in a spacious cage designed to enhance social interaction, supplemented with various toys, tunnels, shelters, and running wheels to promote increased locomotor activity. Compared to standard cages, which typically contain only food and water and fewer mice, an EE offers greater physical, somatosensory, cognitive, and social stimulation possibilities. Exposure to an EE has been shown to induce activity-dependent plasticity, resembling the conditions experienced during human rehabilitation training. Previous studies have demonstrated functional improvements in SCI animal models after exposure to an EE^5–9^, accompanied by various plastic responses, including increased brain-derived neurotrophic factor and presynaptic bouton density^6,7^.

Synaptic plasticity, the ability of synapses to modify their function, is a key component of activity-dependent plasticity. Presynaptic plasticity primarily affects neurotransmitter release by modulating the synaptic vesicle cycle (SVC), particularly fusion and priming processes. Genes associated with the SVC in the presynaptic terminal include *Slc17a6* (neurotransmitter uptake), *Rims1*, *Stxbp1*, *Unc13c*, *Snap25*, *Stx1b* (docking), *Unc13c*, *Snap25*, *Stx1b*, *Cplx1*,*Cplx2* (priming), *Snap25*, *Stx1b* (fusion), and *Dnm1* (endocytosis)^10^. Previous studies reported that spontaneous recovery has been observed in SCI models, with genes such as *Synapsin I, Synaptogyrin, *and *Synaptotagmin*, related to SVC being upregulated^11,12^. In contrast, during the acute phase of SCI, these genes and related genes associated with SVC (such as *SNAP25, Stxbp1,* and *Sv2b*) were downregulated^13–17^.

Furthermore, treadmill training in SCI models has been found to increase gene expression related to postsynaptic plasticity and angiogenesis^18^, and Coyo-Salgado et al. explored the effects of an EE in an SCI model^19,20^. Despite these findings, the effect of EE exposure on presynaptic plasticity in gene expression levels remains unknown.

Therefore, this study aimed to identify a gene expression profile focused on SVC at different time points after SCI using RNA-sequencing and further investigate whether EE could induce presynaptic plasticity by modulating SVC in mice with SCI.

## Materials and methods

All methods were performed in accordance with the relevant guidelines and regulations.

### Animals

Seven-week-old male mice (Orient Bio, Gyeonggi-do, South Korea) were used in this study. CD-1 (ICR) mice were housed in a facility accredited by the Association for Assessment and Accreditation of Laboratory Animal Care for all animal experiments. The experimental procedure was approved by the Institutional Animal Care and Use Committee (IACUC) of Yonsei University Health System (approval number: 2016-0214), and the study adhered to the ARRIVE guidelines.

### Spinal cord contusion

Animals were anesthetized with a mixture of ketamine (100 mg/kg intraperitoneally [IP]) and xylazine (10 mg/kg IP). Following anesthesia, the absence of blinking and withdrawal reflexes was confirmed, and the body temperature was maintained at 37°C within a hypoxic chamber. A dorsal laminectomy was then performed at the ninth thoracic vertebral (T9) level to expose the spinal cord, followed by the administration of a moderate T9 contusive injury using an Infinite Horizons device (Precision Systems and Instrumentation, Lexington, NY, USA) with a moderate force (70 kdyn). The sham group underwent only a dorsal laminectomy without contusive injury. Afterwards, the wound was sutured in layers. Postoperative care included manual bladder expression twice daily until spontaneous voiding returned, typically around ten days post-surgery. To prevent postoperative infection, enrofloxacin (5 mg/kg; Bayrtil, Bayer Korea, Seoul, Korea) was injected subcutaneously twice a day for 5 days. Additionally, meloxicam (5 mg/kg; Metacam, Boehringer Ingelheim, USA) was administered subcutaneously immediately after surgery for pain management.

### RNA preparation

At either 2 or 8 weeks post-operation, the mice were anesthetized with a mixture of ketamine (100 mg/kg IP) and xylazine (10 mg/kg IP). Subsequently, they underwent transcardial perfusion with normal saline to isolate the injured spinal cords. The spinal tissues were then frozen at − 70°C and processed for RNA isolation. Total RNA extraction from the spinal cords of both the SCI and control groups was performed using Trizol reagent (Thermo Fisher Scientific, Waltham, MA, USA) according to the manufacturer’s instructions^21^. The quantity and purity of the extracted RNA were confirmed using a Nanodrop spectrophotometer (Thermo Fisher Scientific).

### RNA-sequencing and transcriptome data analysis

RNA sequencing was performed at Macrogen Inc. (Seoul, South Korea) using the HiSeq 2000 platform (Illumina, San Diego, CA, USA), following previously established protocols^22–24^.

### Enriched KEGG pathway analysis

To identify enriched Kyoto Encyclopedia of Genes and Genomes (KEGG) pathways for differentially expressed genes (DEGs), we used the Database for Annotation, Visualization, and Integrated Discovery (DAVID) 2021 platform (http://david.abcc.ncifcrf.gov/) as previously described^25^.

### Enriched environment

**Figure 1:**
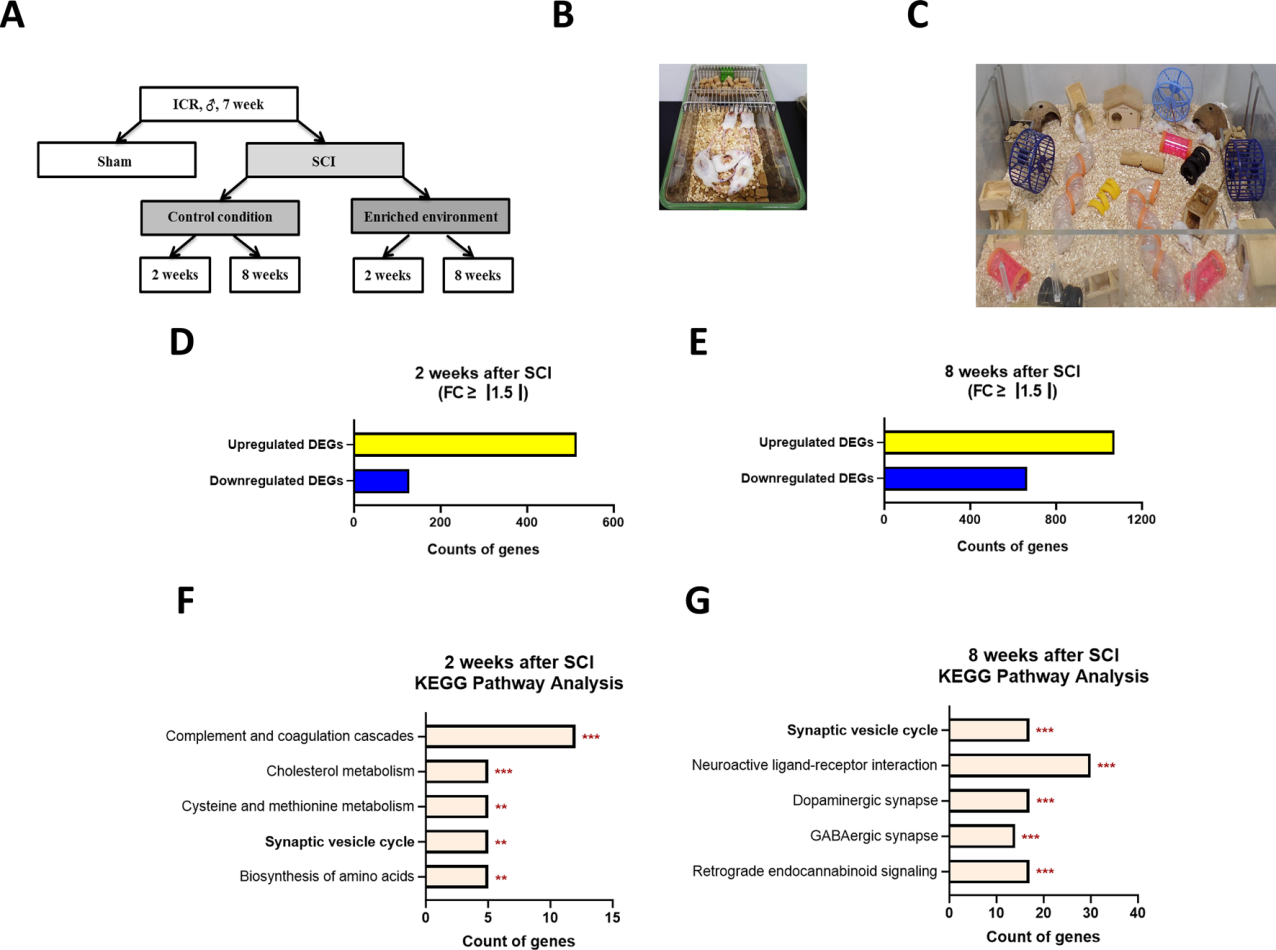
Experimental design and gene expression profile by transcriptome analysis of the SCI compared to the sham group. (**A**) The experimental scheme of this study. Male CD-1 (ICR) mice (7 weeks old) with spinal cord contusion were randomly assigned to a standard (control) or enriched environmental (EE) condition (EE). The control group mice received only a dorsal laminectomy. (**B**) The control group was housed in a standard cage for two or eight weeks. (**C**) The EE group was housed in a large cage containing tunnels, shelters, toys, and running wheels for voluntary exercise, allowing for social interaction. (**D**) The bar graphs show the number of differentially expressed genes (DEGs) with fold change ≥ |1.5| in the SCI group two weeks after SCI. The yellow and blue bars indicate upregulated and downregulated genes, respectively. (**E**) Bar graphs show the number of DEGs with fold change ≥ |1.5| in the SCI group eight weeks after SCI. The yellow and blue bars indicate upregulated and downregulated genes, respectively. (**F**) The top five significant pathways in the SCI group two weeks after SCI. (**G**) The top five significant pathways in the SCI group eight weeks after SCI.

A schematic timeline of this experiment is illustrated in Fig. 1A. The sham group and the control group were housed in standard cages (27 × 22.5 × 14 cm^3^) with three to five mice per cage for either 2 or 8 weeks (Fig. 1B). In contrast, the EE group was housed in larger cages (86 × 76 × 31 cm^3^) for the same durations, which contained novel objects, such as tunnels, shelters, toys, and running wheels to facilitate voluntary exercise and social interaction, with 12–15 mice per cage (Fig. 1C)^26–30^. Notably, the EE cage underwent cleaning twice weekly, at which point the objects and toys were rearranged in order to provide a renewed novel environment.

### Quantitative real-time reverse transcription polymerase chain reaction (qRT-PCR)

Quantitative real-time reverse transcription polymerase chain reaction (qRT-PCR) was employed to validate the transcriptome analysis. Total RNA was reverse-transcribed into complementary DNA (cDNA) using ReverTra Ace® qPCR RT Master Mix with gDNA Remover (Toyobo, Osaka, Japan), according to the manufacturer’s instructions. Messenger RNA (mRNA) expression levels of the genes of interest were profiled using qPCRBIO SyGreen Mix Hi-ROX (PCR BIOSYSTEMS, London, UK) in a StepOnePlus Real-Time PCR System (Applied Biosystems, Foster City, CA, USA). Data analysis was performed using the 2^−ΔΔ*CT*^ method^31^. The primer sequences used for qRT-PCR are detailed in Supplementary Table 1.

### Western blot

For qRT-PCR validation of gene expression, extracted proteins (50 μg) were dissolved in radioimmunoprecipitation assay (RIPA) buffer, boiled for 5 min, and loaded onto Bis–Tris gels (4–12%).The separated proteins were then transferred onto polyvinylidene difluoride membranes (Amersham Pharmacia Biotech, Little Chalfont, UK) using NuPage Transfer Buffer (Invitrogen) containing 20% (vol/vol) methanol at 4°C. The membranes were subsequently blocked for 1 h in Tris-buffered saline (TBS) containing 5% skimmed milk (Difco, BD Biosciences, Oxford, UK), washed thrice with TBS containing 0.01% Tween 20 (TBST) for 10 min each, and incubated overnight at 4 °C with primary antibodies specific to the target proteins. The primary antibodies were SLC176C, STXBP1, SNAP25, DNM1 (1:1000; Abcam, Cambridge, UK), RIM1/2, STX1B, Actin (1:1000; Santa Cruz Biotechnology, Santa Cruz, CA, USA), and CPLX1/2 (1:1000; Cell Signaling Technology, Danvers, MA, USA). On the following day, the blots were washed thrice with TBST and then incubated for 1 h with horse-radish peroxidase-conjugated secondary antibodies (1:4000; Santa Cruz Biotechnology) at room temperature. After re-washing the blots thrice with TBST, they were visualized using an enhanced chemiluminescence detection system (Amersham Pharmacia Biotech, Little Chalfont, UK).

### Immunohistochemistry

The animals were euthanized and perfused transcardially with 4% paraformaldehyde in phosphate buffer (0.1 M; pH 7.4). Subsequently, the spinal cords were post-fixed for 1 h, followed by cryoprotection in sucrose (30%) in TBS containing 0.02% sodium azide. Approximately 1 cm of spinal cord tissue centered on the damaged area was harvested and cryosectioned with a slice thickness of 10 μM along the longitudinal axis. Immunohistochemistry staining was performed on four different sections. For double immunofluorescence labeling, sections were stained with primary antibodies against rabbit anti-SNAP25 (1:400, Abcam), mouse anti-STX1B (1:400, Santacruz), rabbit anti-MAP2 (1:400, Abcam), rabbit anti-GFAP (1:400, Neuromics, USA), rat anti-5-HT (1:400, Abcam), mouse anti- SLC176C (1:400, Abcam), mouse anti- DNM1 (1:400, ThermoFisher), mouse anti- STXBP1 (1:400, LSBio, USA), mouse anti-GFAP (1:400, Abcam), mouse anti-GAP-43 (1:400, Abcam), and secondary antibodies such as Alexa Flour 488 goat anti-rabbit (1:400, Invitrogen) and Alexa Flour 594 anti-mouse (1:400 Invitrogen). The stained sections were mounted on glass slides with a fluorescent mounting medium containing 4′, 6′-diamidino-2-phenylindole (Vectorshield, Vector, Burlingame, CA, USA). Subsequently, the stained sections were analyzed using confocal microscopy (LSM700, Zeiss, Gottingen, Germany), and the integrated density was quantified using ImageJ software.

### Neurobehavior assessments

#### Basso mouse scale (BMS) test

The Basso Mouse Scale (BMS) test is a validated scale used to monitor the progress of hind-limb functional recovery following SCI. The scale ranged from 0 (no ankle movement) to 9 (complete functional recovery). BMS scores were recorded at 3, 7, 14, 28, 42, and 56 days following SCI by two independent examiners blind to the experimental conditions. The hind-limb motion was used to assess coordinated movement and stepping. The average of the two scores was used when differences in the BMS score between the right and left hind limbs were observed.

#### Cylinder rearing test

Typically, when a mouse is introduced into a cylinder, it exhibits spontaneous rearing behavior and uses its forepaws for support. The number of times each forelimb contacted the cylinder wall while the mouse was rearing (Jeung Do B&P, Seoul, Korea) was counted for 5 min.

#### Ladder walking test

To evaluate hind limb function, a horizontal ladder (60 cm) with irregularly spaced metal rungs (Jeung Do B&P) was utilized. Mice were required to traverse the ladder four consecutive times. All trials were video recorded, and the first video was analyzed. The total score in each trial (TS) was determined by summing the obtained scores. Each left and right hind limb was evaluated using the Skilled Walking Performance Score method with some modifications^32–35^. Hindlimb positioning was qualitatively assessed using a foot grading system employing a 7-category scale (0–6 points). This scale, adapted from Metz and Whishaw (2009)^33^ was used to quantify hindlimb position and foot faults observed in placement accuracy, with ratings as follows: 0 (entire miss), 1 (deep slip), 2 (slight slip), 3 (replacement), 4 (correction), 5 (partial placement), and 6 (correct placement).

#### Open field test

The open-field test is utilized to assess the locomotor activity and spontaneous exploration of mice in a novel environment. The test was conducted in a square area measuring 30 × 30 × 30 cm^3^. The floor of the area was divided into 16 sections, with 4 inner sectors representing the center and the 12 outer sectors defined as the periphery. Mice were individually placed into the periphery and allowed to explore freely for 25 min while being monitored with a video camera. Data analyses were performed using the Smart Vision 2.5.21 video tracking system (Panlab, Barcelona, Spain).

### Statistical analysis

All data were expressed as the mean ± standard error of the mean (SEM). Statistical analyses were performed using Statistical Package for Social Sciences (SPSS Inc, Chicago, IL, USA) version 26.0. Comparisons of variables between two groups were analyzed with the Student’s *t*-test, while variables among multiple groups were analyzed using a one-way analysis of variance (ANOVA) followed by a Bonferroni *post-hoc* test. Statistical significance was set at *p* < 0.05.

## Results

### Synaptic vesicle cycle-related genes were differentially expressed genes in SCI

Transcriptome analysis was conducted to identify DEGs during SCI compared to the control group. At 2 weeks post-SCI, 17,155 transcripts were differentially expressed in the SCI group compared to the control group. Among these, 515 transcripts were upregulated by at least 1.5-fold, while 128 transcripts were downregulated by at least -1.5-fold compared to the control group (Fig. 1D, Supplementary Tables 2 and 3). Similarly, at 8 weeks after SCI, 17,048 transcripts were differentially expressed in the SCI group compared to the control group; 1,073 transcripts were upregulated by at least 1.5-fold, and 666 transcripts were downregulated by at least -1.5-fold compared to the control group (Fig. 1E, Supplementary Tables 4 and 5).

Compared to the sham group, up and down-regulated DEGs of SCI at 2 or 8 weeks post-injury were categorized based on enriched KEGG pathways using DAVID 2021. Among the upregulated DEGs at 2 weeks post-SCI, 73 enriched significant pathways (*p* < 0.05) were identified. The top five pathways included mmu04145:Phagosome, mmu05140:Leishmaniasis, mmu05152:Tuberculosis, mmu04640:Hematopoietic cell lineage, and mmu04612:Antigen processing and presentation (Supplementary Table 6). Similarly, among the upregulated DEGs at 8 weeks post-SCI, 84 enriched significant pathways (*p* < 0.05) were identified, with the top five pathways being mmu04145:Phagosome, mmu04380:Osteoclast differentiation, mmu04610:Complement and coagulation cascades, mmu05140:Leishmaniasis, and mmu05152:Tuberculosis (Supplementary Table 7). However, these upregulated DEGs in enriched significant pathways were not directly related to SCI. Thus, we decided to focus on down-regulated DEGs of enriched significant pathways.

Among the down-regulated DEGs at 2 weeks post-SCI, 17 enriched significant pathways (*p* < 0.05) were identified (Supplementary Table 8). The top five pathways included mmu04610:Complement and coagulation cascades, mmu04979:Cholesterol metabolism, mmu00270:Cysteine and methionine metabolism, mmu04721:Synaptic vesicle cycle, and mmu01230:Biosynthesis of amino acids, as shown in Fig. 1F. Statistically significant pathways (*p* < 0.01) are listed in Table 1.

**Table 1:** Enriched KEGG pathways were identified to be significantly down-regulated in 2 weeks post-SCI.

Term	Count	p-value	Genes	Fold Enrichment
mmu04610:Complement and coagulation cascades	12	1.36E-11	FGB, SERPINA1A, FGA, SERPINA1B, SERPINA1C, SERPINA1D, FGG, PLG, SERPINA1E, F2, KNG1, MBL2	19.526
mmu04979:Cholesterol metabolism	5	2.85E-04	APOA2, APOC3, APOA1, APOA4, LDLR	15.295
mmu00270:Cysteine and methionine metabolism	5	4.13E-04	BHMT, SDS, TAT, MAT1A, GNMT	13.905
**mmu04721:Synaptic vesicle cycle**	**5**	**0.001**	**SLC6A5, RIMS1, STX1B, SLC17A6, DNM1**	**9.932**
mmu01230:Biosynthesis of amino acids	5	0.002	SDS, CPS1, ARG1, ALDOB, MAT1A	9.680
mmu05143:African trypanosomiasis	4	0.002	PLCB4, APOA1, HBA-A2, KNG1	15.687
mmu01100:Metabolic pathways	21	0.002	CAR3, SDS, ARG1, TAT, RDH7, CYP3A11, MAT1A, GNMT, SELENBP2, ADH1, BHMT, PLCB4, TDO2, CPS1, ALDH1A2, UGT2B5, ALDOB, PCK1, FBP1, HPD, UOX	1.981
mmu03320:PPAR signaling pathway	5	0.003	FABP1, APOA2, APOC3, APOA1, PCK1	8.593
mmu00830:Retinol metabolism	5	0.003	ADH1, ALDH1A2, RDH7, CYP3A11, UGT2B5	7.884
mmu04611:Platelet activation	5	0.008	FGB, FGA, PLCB4, FGG, F2	6.118
mmu00010:Glycolysis/Gluconeogenesis	4	0.009	ADH1, ALDOB, PCK1, FBP1	9.131

These pathways are statistically significant (*p* < 0.01).

Synaptic vesicle cycle pathway is shown in a bold font.

Similarly, among the down-regulated DEGs at 8 weeks post-SCI, 55 enriched pathways (*p* < 0.05) were identified (Supplementary Table 9). The top five pathways included mmu04721:Synaptic vesicle cycle, mmu04080:Neuroactive ligand-receptor interaction, mmu04728:Dopaminergic synapse, mmu04727:GABAergic synapse, and mmu04723:Retrograde endocannabinoid signaling, as depicted in Fig. 1G. Statistically significant pathways (*p* < 0.001) are presented in Table 2.

**Table 2:** Enriched KEGG pathways were identified to be significantly down-regulated in 8 weeks post-SCI.

Term	Count	*p*-value	Genes	Fold enrichment
**mmu04721:Synaptic vesicle cycle**	**17**	**3.98E−11**	**NSF, UNC13C, SNAP25, SLC32A1, STXBP1, SLC1A2, SLC6A11, CPLX2, CPLX1, DNM1, SLC6A5, DNM3, RIMS1, STX1B, ATP6V1B2, SLC17A6, SLC18A3**	**8.917**
mmu04080:Neuroactive ligand-receptor interaction	30	9.47E−08	NPSR1, CHRNA4, OPRL1, HTR2C, GRM1, GLRA2, APELA, HTR7, CNR1, GRM8, DRD2, PAQR9, GABRA1, GABBR2, LYNX1, NPY5R, GRID1, GABRA3, HTR1A, TACR1, ADRA2C, SSTR2, GABRG2, TRHR, APLN, UTS2B, GLRB, CCKBR, NMS, LYPD6B	3.099
mmu04728:Dopaminergic synapse	17	1.89E−07	KCNJ9, MAPK10, MAPK9, MAPK8, PLCB4, GNAL, TH, PPP2R2B, GNG4, KIF5C, AKT3, KIF5A, CALM3, DRD2, CALM2, KCNJ3, SCN1A	5.086
mmu04727:GABAergic synapse	14	2.37E−07	NSF, GABRA1, GABBR2, SLC12A5, SLC32A1, GAD1, GABRA3, ADCY3, GAD2, SLC6A11, ABAT, ADCY1, GABRG2, GNG4	6.353
mmu04723:Retrograde endocannabinoid signaling	17	6.72E−07	GABRA1, SLC32A1, KCNJ9, GABRA3, ADCY3, ADCY1, GRM1, GABRG2, MAPK10, RIMS1, MAPK9, MAPK8, PLCB4, CNR1, GNG4, SLC17A6, KCNJ3	4.639
mmu04261:Adrenergic signaling in cardiomyocytes	16	6.59E−06	CACNA2D3, ATP2B3, ATP1A3, ADCY3, ATP2B2, ADCY1, ATP1B1, PLCB4, PPP2R2B, TNNT2, AKT3, CALM3, CACNG2, CALM2, SCN4B, RAPGEF4	4.143
mmu04024:cAMP signaling pathway	17	1.30E−04	GABBR2, HTR1A, ATP2B3, ATP1A3, ADCY3, ATP2B2, ADCY1, ATP1B1, SSTR2, MAPK10, MAPK9, MAPK8, AKT3, CALM3, DRD2, CALM2, RAPGEF4	3.065
mmu04925:Aldosterone synthesis and secretion	11	2.00E−04	CACNA1I, PLCB4, PRKCE, ATP2B3, ATP1A3, ADCY3, ATP2B2, CALM3, ADCY1, ATP1B1, CALM2	4.356
mmu04072:Phospholipase D signaling pathway	13	3.05E−04	DGKG, SHC3, ADCY3, ADCY1, GRM1, DNM1, DNM3, PLCB4, GRM8, AKT3, PIP5K1B, DGKK, RAPGEF4	3.524
mmu05032:Morphine addiction	10	3.89E−04	GABRA1, GABBR2, SLC32A1, GNG4, KCNJ9, GABRA3, ADCY3, ADCY1, GABRG2, KCNJ3	4.438
mmu04915:Estrogen signaling pathway	12	4.32E−04	GABBR2, SHC3, PLCB4, KCNJ9, AKT3, ADCY3, CALM3, PGR, ADCY1, CALM2, GRM1, KCNJ3	3.644
mmu04971:Gastric acid secretion	9	4.91E−04	PLCB4, CCKBR, ATP1A3, ADCY3, CALM3, ADCY1, ATP1B1, SSTR2, CALM2	4.847
mmu04020:Calcium signaling pathway	17	5.13E−04	ATP2B3, HTR2C, ADCY3, ATP2B2, ADCY1, TACR1, GRM1, TRHR, CACNA1I, HTR7, PLCB4, GNAL, SLN, CCKBR, FGF18, CALM3, CALM2	2.714
mmu05231:Choline metabolism in cancer	10	6.73E−04	DGKG, MAPK10, MAPK9, SLC5A7, MAPK8, PDPK1, AKT3, PIP5K1B, DGKK, WASF3	4.121
mmu04713:Circadian entrainment	10	6.73E−04	CACNA1I, PLCB4, GNG4, KCNJ9, NOS1AP, ADCY3, CALM3, ADCY1, CALM2, KCNJ3	4.121
mmu04929:GnRH secretion	8	8.64E−04	CACNA1I, GABBR2, PLCB4, KCNJ11, KCNJ9, AKT3, KCNJ3, HCN1	5.129
mmu05142:Chagas disease	10	9.65E−04	MAPK10, MAPK9, MAP2K4, MAPK8, GNAL, PLCB4, PPP2R2B, AKT3, ADCY1, CALR4	3.921

These pathways are statistically significant (*p* < 0.001).

Synaptic vesicle cycle pathway is shown in a bold font.

The ‘SVC pathway,’ which was the focus of our study, exhibited downregulation in the SCI group at both 2 and 8 weeks post-injury compared to the control group (Fig. 1F,G). Specifically, at 2 weeks after SCI, genes such as *SLC6A5*, *RIMS1*, *STX1B*, *SLC17A6*, and *DNM1* were significantly downregulated compared to the control group. Similarly, at 8 weeks post-SCI, genes including *NSF*, *UNC13C*, *SNAP25*, *SLC32A1*, *STXBP1*, *SLC1A2*, *SLC6A11*, *CPLX2*, *CPLX1*, *DNM1*, *SLC6A5*, *DNM3*, *RIMS1*, *STX1B*, *ATP6V1B2*, *SLC17A6*, and *SLC18A3* were significantly downregulated compared to the control group. Notably, *SLC6A5*, *RIMS1*, *STX1B*, *SLC17A6*, and *DNM1* consistently downregulated at both 2 and 8 weeks post-SCI compared to the control group.

### Exposure to EE-regulated SVC in SCI

**Figure 2:**
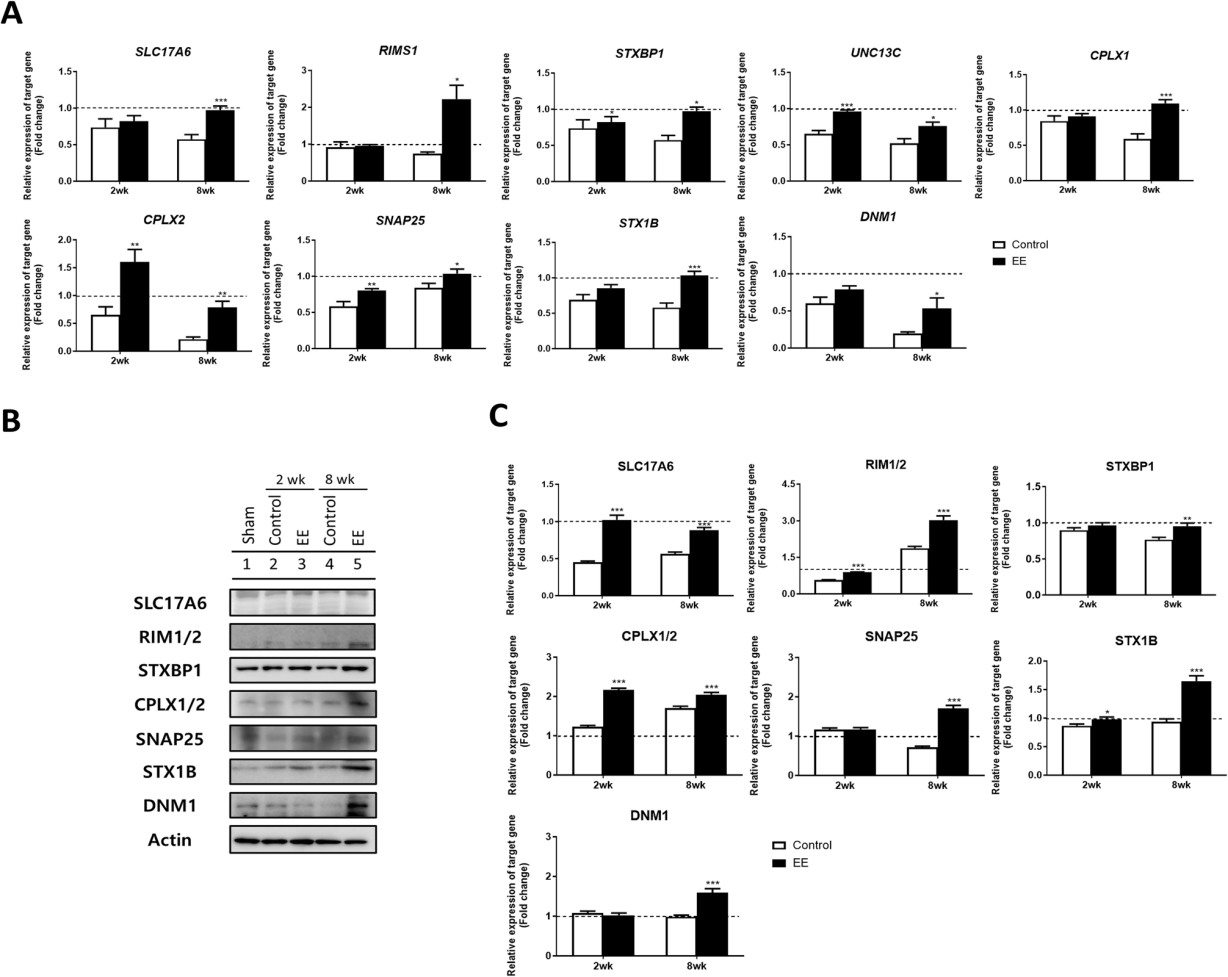
EE exposure regulated SVC-related genes after SCI. (**A**) The relative expression of target genes for quantitative real-time reverse transcription polymerase chain reaction (qRT-PCR) was calculated using the 2^*−∆∆Ct*^ method. All results are expressed as the mean ± standard error of the mean (SEM). The white box indicates the SCI-Control group and the black box indicates the SCI-EE group. The expression of target genes was relative to the control. The expression values are 1 and represented in the graph as a dotted line. Control group (n = 4); two weeks SCI-SC group (n = 4); two weeks SCI-EE group (n = 4); eight weeks SCI-SC group (n = 4); eight weeks SCI-EE group (n = 4). (**B**) Western blot analysis was performed using antibodies against SLC17A6, RIM1/2, STXBP1, CPLX1/2, SNAP25, STX1B, DNM1, and Actin (control). (**C**) Comparison of relative protein expression of SCI mice after exposure to EE (EE) or standard cages (control) for two and eight weeks compared to the control group. All results are expressed as the mean ± SEM. Control group (n = 4); two weeks SCI-SC group (n = 4); two weeks SCI-EE group (n = 4); eight weeks SCI-SC group (n = 4); eight weeks SCI-EE group (n = 4). All results are expressed as means ± standard error of the mean (SEM). *P < 0.05, **P < 0.01 and ***P < 0.001.

The expression levels of target genes, such as *SLC17A6*, *RIMS1*, *STXBP1*, *UNC13C*, *CPLX1*, *CPLX2*, *SNAP25*, *STX1B,* and *DNM1,* related to SVC, were validated through qRT-PCR in both the SCI-EE and SCI-Control groups at 2 and 8 weeks compared to the control (Fig. 2A). Target gene expression was normalized to *GAPDH* expression and presented relative to the control group (indicated by a dotted line and the expression value as 1.0 in the graph).

In the SCI-Control group at 2 weeks post-injury, the expression of the target genes decreased compared to the control group, with fold changes as follows: *SLC17A6* (0.736-fold), *RIMS1* (0.917-fold), *STXBP1* (0.584-fold), *UNC13C* (0.651-fold), *CPLX1* (0.843-fold), *CPLX2* (0.654-fold), *SNAP25* (0.584-fold), *STX1B* (0.688-fold), and *DNM1* (0.602-fold). Conversely, in the SCI-EE group at 2 weeks post-injury, the expression of the target genes increased compared to the SCI-Control group, with fold changes as follows: *SLC17A6* (0.821-fold), *RIMS1* (0.954-fold), *STXBP1* (0.938-fold), *UNC13C* (0.960-fold), *CPLX1* (0.908-fold), *CPLX2* (1.062-fold), *SNAP25* (0.805-fold), *STX1B* (0.855-fold), and *DNM1* (0.788-fold). Specifically, *STXBP1*, *UNC13C*, *CPLX2,* and *SNAP25* in the SCI-EE group exhibited significant fold changes compared to the SCI-Control group at 2 weeks post-injury, with *UNC13C* (*p* < 0.001), *CPLX2* and *SNAP25* (*p* < 0.01), and *STXBP1* (*p* < 0.05).

Furthermore, at 8 weeks post-injury, the expression of the target genes decreased in the SCI-Control group compared to the control group, with fold changes as follows: *SLC17A6* (0.574-fold), *RIMS1* (0.741-fold), *STXBP1* (0.741-fold), *UNC13C* (0.522-fold), *CPLX1* (0.588-fold), *CPLX2* (0.216-fold), *SNAP25* (0.839-fold), *STX1B* (0.581-fold), and *DNM1* (0.196-fold). In contrast, the expression of the target genes increased in the SCI-EE group at 8 weeks compared to the SCI-Control group, with fold changes as follows: *SLC17A6* (0.973-fold), *RIMS1* (2.221-fold), *STXBP1* (1.042-fold), *UNC13C* (0.757-fold), *CPLX1* (1.093-fold), *CPLX2* (0.786-fold), *SNAP25* (1.037-fold), *STX1B* (1.036-fold), and *DNM1* (0.535-fold). All genes in the SCI-EE group exhibited significant fold changes compared to the SCI-Control group at 8 weeks, with *SLC17A6*, *CPLX1*, and *STX1B* (*p* < 0.001), *CPLX2* (*p* < 0.01), and *RIMS1*, *STXBP1*, *UNC13C*, *SNAP25*, and *DNM1* (*p* < 0.05).

The expression of SLC17A6*,* RIM1/2*,* STXBP1*,* CPLX1/2*,* SNAP25*,* STX1B*,* and DNM1 was further validated using western blotting in SCI mice exposed to EE or standard cages for 2 and 8 weeks, compared to the control group (Fig. 2B,C). The gene expression levels were normalized to Actin expression and presented relative to the control group (indicated by dotted lines and the protein value represented as 1.0 in the graph). At 2 weeks post-injury, the relative protein levels in the SCI-Control group were as follows: SLC17A6 (0.452-fold), RIM1/2 (0.571-fold), STXBP1 (0.899-fold), CPLX1/2 (1.226-fold), SNAP25 (1.163-fold), STX1B (0.864-fold), and DNM1 (1.075-fold). In contrast, the relative protein levels in the SCI-EE group at 2 weeks were: SLC17A6 (1.025-fold), RIM1/2 (0.890-fold), STXBP1 (0.964-fold), CPLX1/2 (2.165-fold), SNAP25 (1.174-fold), STX1B (0.982-fold), and DNM1 (0.983-fold). Specifically, the protein levels of SLC17A6, RIM1/2, CPLX1/2, and STX1B were significantly higher in the SCI-EE group compared to the SCI-Control group at 2 weeks, with SLC17A6, RIM1/2, and CPLX1/2 (*p* < 0.001), and STX1B (*p* < 0.05).

At 8 weeks post-injury, the relative protein levels in the SCI-Control group were as follows: SLC17A6 (0.563-fold), RIM1/2 (1.873-fold), STXBP1 (0.767-fold), CPLX1/2 (1.707-fold), SNAP25 (0.715-fold), STX1B (0.938-fold), and DNM1 (1.023-fold). Conversely, the relative protein levels in the SCI-EE group at 8 weeks were: SLC17A6 (0.883-fold), RIM1/2 (3.030-fold), STXBP1 (0.953-fold), CPLX1/2 (2.064-fold), SNAP25 (1.700-fold), STX1B (1.648-fold), and DNM1 (1.594-fold). All protein levels were significantly higher in the SCI-EE group than in the SCI-Control group at 8 weeks (*p* < 0.001).

**Figure 3:**
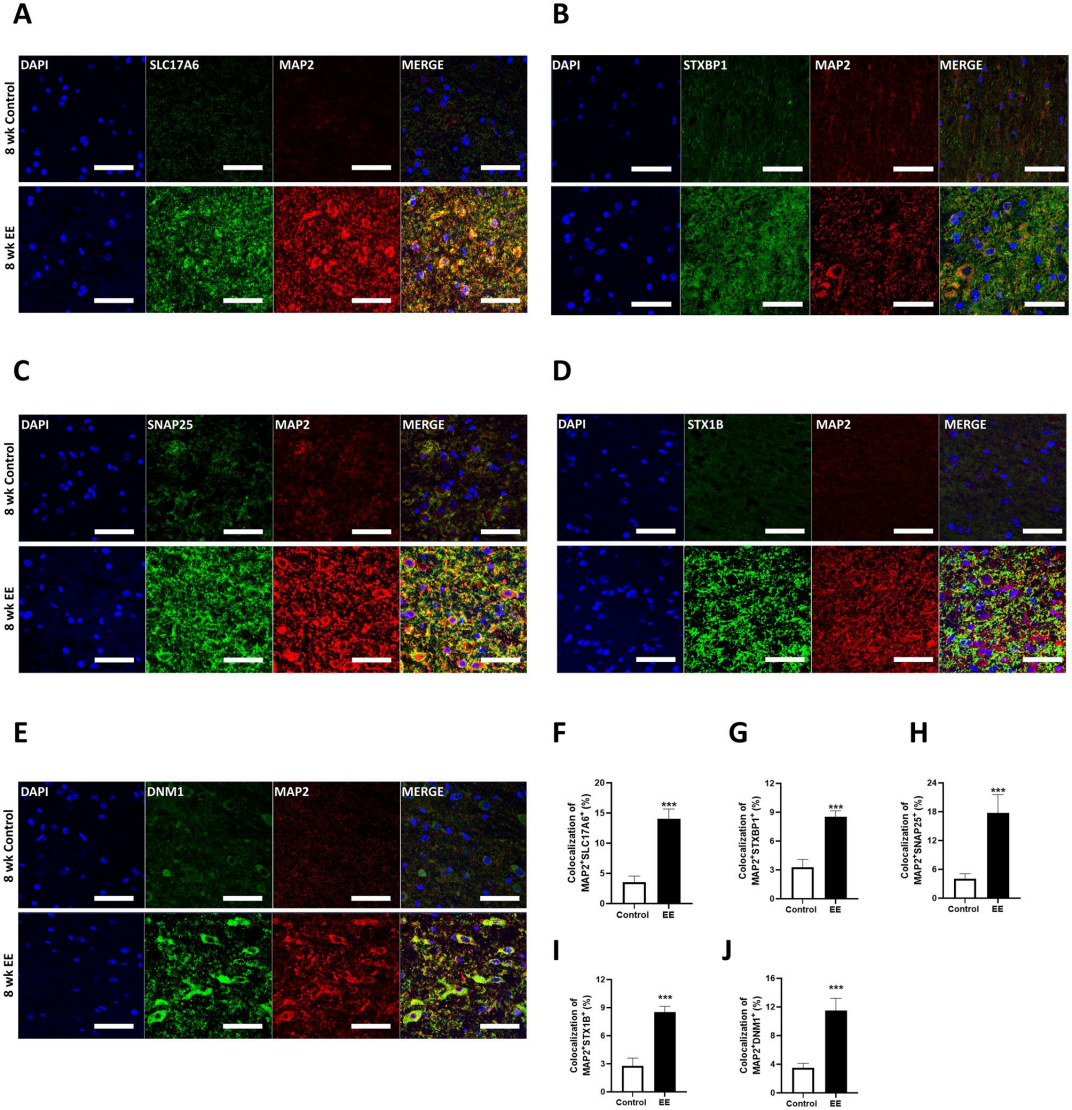
Prolonged EE exposure increased SVC-related genes. (**A**) Representative confocal microscopic images of MAP2^+^SLC17A6^+^ in the spinal cord. (**B**) Representative confocal microscopic images of MAP2^+^STXBP1^+^ in the spinal cord. (**C**) Representative confocal microscopic images of MAP2^+^SNAP25^+^ in the spinal cord. (**D**) Representative confocal microscopic images of MAP2^+^STX1B^+^ in the spinal cord. (**E**) Representative confocal microscopic images of MAP2^+^DNM1^+^ in the spinal cord. Scale bar : 50 μM. (**F**–**J**) The percentage of colocalization with MAP2 and SLC17A6, STXBP1, SNAP25, STX1B, and DNM1, respectively in the spinal cord. 8 weeks SCI SC group (N = 3); 8 weeks SCI EE group (N = 3); Centering on the damaged area, an approximately 1 cm length of spinal cord tissue. All results are expressed as means ± standard error of the mean (SEM). ***P < 0.001.

To examine the synaptic vesicle pathway in SCI after exposure to EE for 8 weeks compared to standard cages, histological assessments with MAP2, STX1B, SNAP25, SLC17A6, DNM1, and STXBP1 were conducted. Representative images of immunohistochemistry results are shown in Fig. 3A–E. In SCI mice exposed to EE for 8 weeks compared to those in standard cages for the same duration, colocalizations of MAP2 with STX1B, SNAP25, SLC17A6, DNM1, and STXBP1, were significantly increased, respectively (n = 4). These findings suggest that prolonged EE exposure induces SVC-related genes in SCI.

### Exposure to EE-regulated neurobehavior assessments.

**Figure 4:**
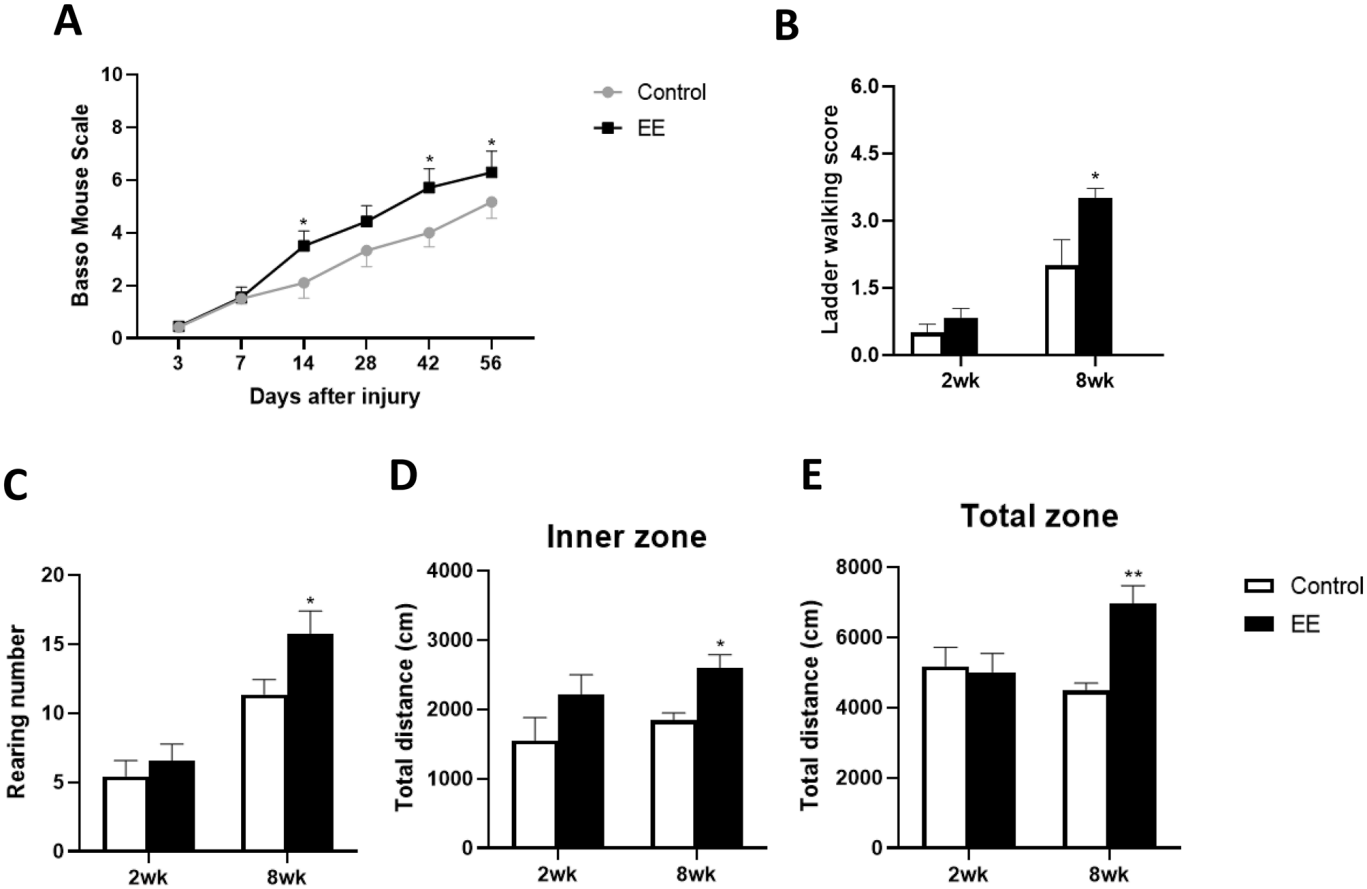
EE exposure improved neurobehavior function. (**A**) The Basso Mouse Scale (BMS) scores were obtained from day 3 to day 56 post-injury. SCI-SC group (n = 6); SCI-EE group (n = 7) (**B**) Ladder walking score in SCI-EE compared to SCI-Control at 2 and 8 weeks. 2 weeks SCI SC group (N = 4); 2 weeks SCI EE group (N = 5); 8 weeks SCI SC group (N = 3); 8 weeks SCI EE group (N = 3) (**C**) In the cylinder rearing test, the rearing count in the SCI-EE group was compared to that of the SCI-Control group at two and eight weeks. Two weeks SCI-SC group (n = 4); two weeks SCI-EE group (n = 4); eight weeks SCI-SC group (n = 4); eight weeks SCI-EE group (n = 5) (**D**) Total zone distance of the first interval in the SCI-EE group compared to the SCI-Control group at 2 and 8 weeks. Two weeks SCI-SC group (n = 4); Two weeks SCI-EE group (n = 5); Eight weeks SCI SC group (n = 4); eight weeks SCI-EE group (n = 5) (**E**) Inner zone distance of the first interval in the SCI-EE group compared to in the SCI-Control group at two and eight weeks. Two weeks SCI-SC group (n = 4); 2 weeks SCI-EE group (n = 5); 8 weeks SCI SC group (n = 4); eight weeks SCI EE group (n = 5); All results are expressed as means ± standard error of the mean (SEM). *P < 0.05, **P < 0.01 and ***P < 0.001.

To evaluate whether exposure to EE for 8 weeks could induce functional recovery in SCI compared to standard cages, several tests were conducted, including the BMS, ladder walking test, cylinder test, and Open-field test. The mean BMS scores gradually increased after 3 days post-injury, with significant differences observed at 14, 42, and 56 days post-injury, where the SCI-EE group showed significantly higher scores than the SCI-Control group (Fig. 4A). Similarly, in the ladder walking test, hindlimb scale scores were progressively higher in the SCI-EE group at 8 weeks compared to the SCI-Control group (Fig. 4B), indicating improved locomotor functional recovery with prolonged EE exposure following SCI.

In the cylinder rearing test, the rearing count was significantly higher in the SCI-EE group at 8 weeks compared to the SCI-Control group (Fig. 4C). Moreover, in the open-field test, both total and inner zone distances were significantly increased in the SCI-EE group at 8 weeks compared to the SCI-Control group (Fig. 4D,E). Taken together, these results suggest that prolonged EE exposure promotes locomotor functional recovery in SCI mice.

### Exposure to EE-regulated axon regeneration in SCI

Serotonin (5-HT) has been recognized for its role in promoting axonal regeneration and repair in SCI^36^. Additionally, growth-associated protein, GAP-43, is crucial for axonal regeneration in damaged neurons^37^. Glial fibrillary acidic protein (GFAP) is well-established marker for glial scar formation, an indicator of wound healing after SCI^38^. Here, we analyzed GFAP in conjunction with 5-HT and GAP-43 as an indicator of glial scar formation and axonal regeneration, respectively.

**Figure 5:**
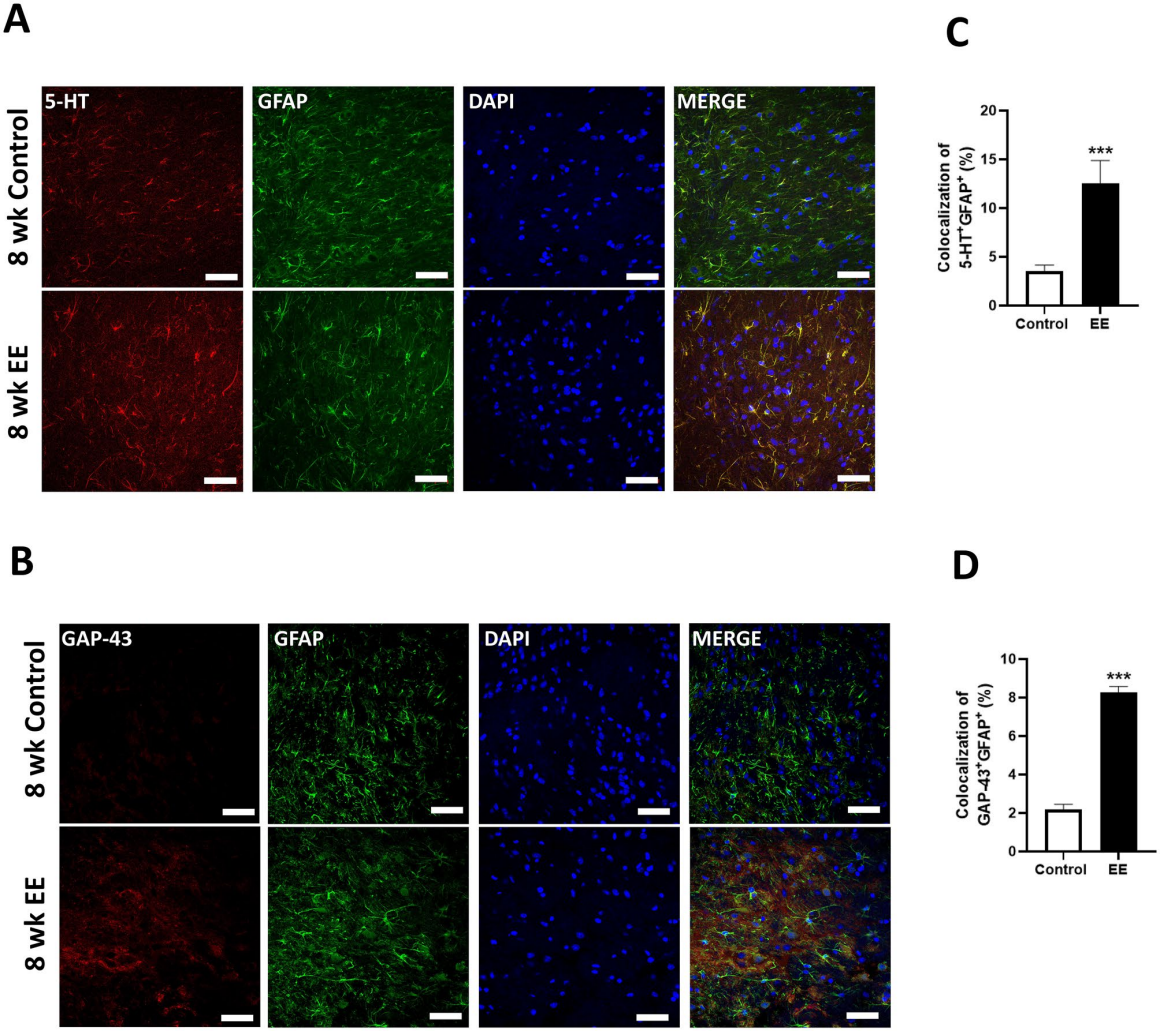
Prolonged EE exposure promoted axon regeneration after SCI. (**A**) Representative confocal microscopic images of GFAP^+^5-HT^+^ cells in the spinal cord. (**B**) Representative confocal microscopic images of GFAP^+^GAP-43^+^ cells in the spinal cord. Scale bar: 50 μM. (**C**, **D**) The percentage of colocalization with GFAP with 5-HT and GAP-43, respectively in the spinal cord. 8 weeks SCI SC group (N = 3); 8 weeks SCI EE group (N = 3); Centering on the damaged area, an approximately 1 cm length of spinal cord tissue. All results are expressed as means ± standard error of the mean (SEM). *** *P* < 0.001.

Histological assessments involving GFAP, 5-HT, and GAP43 were conducted to determine if exposure to EE for 8 weeks could facilitate repair in SCI compared to standard cages. The representative immunohistochemistry results are illustrated in Fig. 5A,B. Comparing SCI mice exposed to EE for 8 weeks with those in standard cages for the same duration, we observed a significant increase in the colocalization of GFAP with 5-HT and GAP43, respectively (n = 4). These findings suggest that prolonged EE exposure promotes axon regeneration in SCI.

## Discussion

In this study, we examined the gene expression profile at various time points after SCI and evaluated the therapeutic potential of EE in an SCI mouse model. Notably, we identified several enriched pathways in DEGs that were either upregulated or downregulated at 2- and 8-weeks post-SCI. Among enriched pathways, the SVC pathway was found to be downregulated in the SCI group at both 2- and 8-weeks post-injury compared to the control group. The downregulation of SVC-related genes in SCI mice suggests a decrease in synaptic function following SCI compared to the control group. Therefore, we specifically focused on investigating changes in presynaptic plasticity following EE exposure in a mouse model of SCI.

Gene expression was notably higher in the SCI-EE group compared to the SCI-Control group at both 2- and 8-weeks post-SCI. Particularly, at 8 weeks after SCI, genes exhibited significantly higher expression levels in the SCI-EE group compared to the SCI-Control group. Moreover, at 8 weeks post-SCI, *RIMS1*, *STXBP1*, *CPLX1*, *SNAP25*, and *STX1B* showed higher gene expression in the SCI-EE group compared to the control group. These results suggest that exposure to EE modulated the SVC in SCI mice, with more pronounced changes observed with prolonged exposure to EE.

Importantly, the observed statistical differences translated into functional improvements in the SCI-EE group, which was confirmed through neurobehavioral assessments including the BMS, ladder walking test, cylinder rearing, and open field test. The EE group demonstrated significant recovery compared to the SC group post-SCI. Specifically, compared to the SC group, the EE group showed greater accuracy in hindlimb placement in the ladder walking test, rearing test, open field test, and BMS scale. Here, it serves to note that as previous studies report a partial spontaneous recovery of locomotor activities in rodent models several weeks after a spinal cord injury^39^, our data indicates increased recovery when exposed to EE regardless of any potential spontaneous locomotion recovery. Additionally, previous studies reported data on SCI mouse models divided into three groups by different functional prognoses: 50 kdyn (mild SCI model), 70 kdyn (moderate SCI model) and 90 kdyn (severe SCI model), induced via a spinal cord impactor^40^. Accordingly, in our study, we evaluated the effects of EE in mice beginning three days after a moderate degree of spinal cord injury. Early phase EE exposure has been shown to substantially improve locomotor outcomes after moderate SCI in rodent models^41^. Concurrently, our study also indicated improved recovery following moderate SCI when supplemented with EE exposure, compared to control mice.

As recovery of locomotion post-SCI can be attributed, at least in part, to axonal regeneration, we sought to interrogate the extent of these processes via immunohistochemical assays targeting axonal growth and regeneration markers. Both the neurotransmitter serotonin (5-HT) and GAP-43 expression have been shown to indicate controlled axon regeneration in spinal cord injury^42^. Notably, EE exposure led to increased 5-HT and GAP-43 immunoreactivity compared to the control group, suggesting that axon regeneration may contribute to improvements in locomotion post-SCI^19,43^. In the case of GFAP, glial fibrillary acidic protein (GFAP) is well-established marker for glial scar formation. While there is still much controversy over the role of glial scar formation, as it is known to have both beneficial and potentially suppressive roles in axonal generation after SCI^38^, it is a well-established marker of wound healing. Thus, in this study, when observed in conjunction with axonal regenerative markers, we have interpreted the presence of GFAP as associating positively with lesion repair.

Here we note that as our research did not interrogate in-depth the origin of the axonal regeneration, we cannot definitively say that the origin of the axonal growth was due to the induction of synaptic plasticity. Indeed, future research into the mechanisms by which presynaptic plasticity, EE, and axonal regeneration is induced is needed to confirm the relationship between these processes.

Based on these results, it can be inferred that EE exposure enhances presynaptic plasticity by modulating gene expression related to the SVC as well as supporting axonal regrowth during the recovery phase. Presynaptic plasticity involves the modification of neurotransmitter release through modulation of processes such as synaptic vesicle fusion and priming, with crucial changes in the size of the primed vesicle pool^44^. The far-reaching effects of presynaptic plasticity emphasize the importance of processes occurring pre-secretion and fusion in the SVC. Additional forms of synaptic plasticity, notably postsynaptic plasticity, occur through changes in synaptic formation, including alterations in the number, shape, and size of dendritic spines as well as in the organization of the postsynaptic membrane^45^. As our study focuses mainly on the role of EE in promoting changes in the SVC leading to pre-synaptic plasticity, we have not significantly explored potential alterations in post-synaptic plasticity following EE exposure after SCI. Currently, there are many studies supporting a potential role for post-synaptic plasticity in encouraging axonal regeneration and recovery. Notably, recent studies have shown that following SCI, electrical stimulation can significantly increase the growth and branching of axons via upregulation of axon growth- and regeneration-promoting genes and factors, which also contributes to induction of Hebbian plasticity^46,47^. Furthermore, previously “silent” (NDMA-only) synapses have been found to undergo activation and reorganization in the face of electrical stimulation, which has been proposed to be a potential mechanism for rejuvenating neural pathways via encouragement of post-injury synaptic plasticity and axonal regrowth^46^.

This may provide a new avenue for patient treatment when in conjunction with locomotion therapy. While locomotion therapy for SCI patients is known to encourage regeneration of neurons, future studies which further determine the extent and mechanism of both pre- and post-synaptic plasticity after supplementary exposure to EE will provide additional valuable information to improve strategies for rehabilitation.

Our data reveals modulation of SVC-associated gene expression by exposure to EE after SCI. Future work remains to be done in determining the cells and processes involved in promoting neural regeneration as a whole. For example, recent research has found potential in the therapeutic use of induced neural stem cells in promoting neuronal regrowth and regeneration following traumatic spinal injury^48^. While iPSCs or induced NSCs have significant potential for therapeutic use, many of the body’s own cell types can contribute to promoting neuroplasticity. The spinal cord is comprised of two main neuronal populations known as spinal interneurons and projection neurons. Spinal interneurons (SpINs) comprise a wide range of neuronal types such as (1) long and short propriospinal neurons, with ascending and descending projections; and (2) local SpINs with either ipsilateral projections, which do not cross the spinal midline, or commissural projections, which do. With their cell body in the spinal cord and projections reaching toward other parts of the CNS or periphery, these neurons have strong potential to contact other cells^49^. While this feature of SpINs makes them well-suited to regulation of motor, sensory, autonomic networks in the CNS, it also allows these neuronal populations to be far-reaching key effectors of plasticity and recovery. Concurrently, activation of these neurons by electrical stimulation, epidural treatment, and activity-based therapies (similar to EE) following SCI has demonstrated increased intraneuronal activity and patient improvement^49^. For this reason, approaches combining EE with targeted activity or stimulation of specific neuronal populations such as SpINs might offer a more personalized and effective treatment to encourage neuroplasticity. Induction of other supporting neural cell populations or factors via electrical or drug-based interventions may also provide an optimal environment to support neuronal plasticity, especially when supplemented with EE, though research in this area is still in its infancy. Additionally, deeper mechanistic studies identifying local and systemic pathways contributing to restoration of neurons after SCI will increase our understanding of the processes underlying neuronal plasticity in the CNS and provide a basis for improved therapeutic strategies.

**Figure 6:**
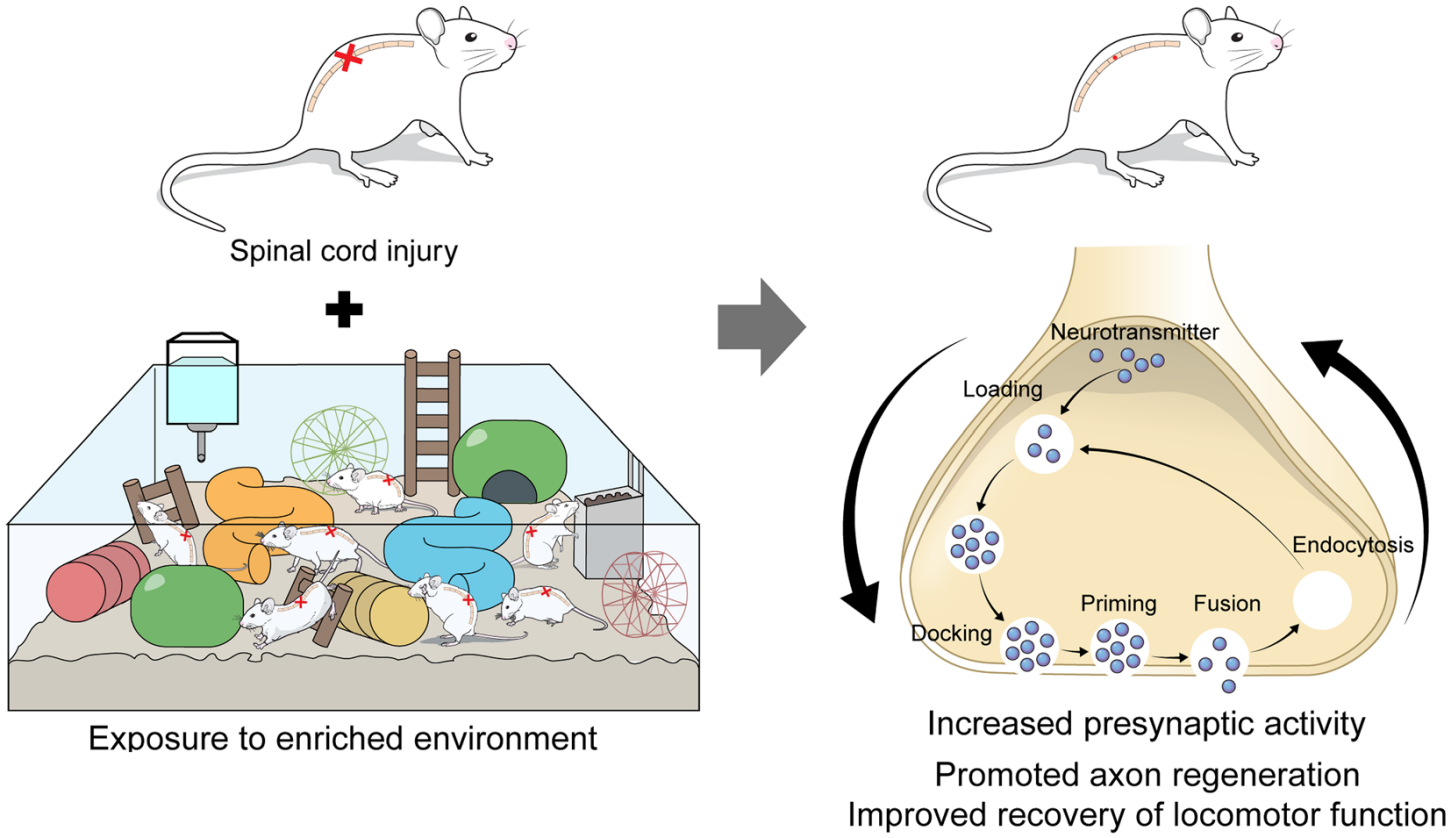
Scheme of the exposure to an EE modulates the SVC in mice with SCI. Exposure to an EE is a suitable method for inducing presynaptic activity, axon regeneration, and neurobehavior function in a mouse spinal cord injury model. These results suggested that prolonged EE exposure may further activate synaptic plasticity.

In conclusion (Fig. 6), our data demonstrates that exposure to an EE modulates the SVC in mice with SCI with potentially therapeutic applications. We also propose that presynaptic plasticity might play a role in promoting axon regeneration and functional improvements in the EE group. We additionally showed that exposure to an EE is a suitable method for inducing activity-dependent plasticity, which may provide similar therapeutic effects during rehabilitation of SCI patients.

### Supplementary Information

Supplementary Table 1.

Supplementary Table 2.

Supplementary Table 3.

Supplementary Table 4.

Supplementary Table 5.

Supplementary Table 6.

Supplementary Table 7.

Supplementary Table 8.

Supplementary Table 9.

## Data Availability

RNA-Seq data used in this study are deposited in the NCBI database as the sequence read archive (SRA) format under the accession number PRJNA945506.
