# Supplementary material for: Exposure to an enriched environment modulates the synaptic vesicle cycle in a mouse spinal cord injury model
Source: Sci Rep. 2024 May 25;14:11946. doi: 10.1038/s41598-024-62112-0 (PMC11126684; doi:10.1038/s41598-024-62112-0)
Supplement: Supplementary file 1 — Supplementary Table 1. [file 41598_2024_62112_MOESM1_ESM.docx]

**Supplementary Table 1. Primers used for qRT-PCR**

| Gene symbol | Forward primer (5’→3’) | Reverse primer (5’→3’) |
| --- | --- | --- |
| *SLC17A6* | GCT GGA AAA TCC CTC GGA CA | GCA TAG CGG AGC CTT CT |
| *RIMS1* | CCA GAG CAA AAC GAG GAC GA | TTG TCG GTG CGT CCT TTC TC |
| *STXBP1* | CGG TCC CCG CCT CAT TAT TT | GCA GTT TCT GTG GGG TGA GA |
| *UNC13C* | TGG GAA AGA GCT AGA CCC TGA | CGC ACT GTT CTT ATT CG |
| *CPLX1* | AAG TAC GCC AAG ATG GAG GC | GGG ATA GCC TTC TTG GGT CG |
| *CPLX2* | AGC CCT GGA ACA GCC CT | GCG GCC CTG GCA GAT ATT |
| *SNAP25* | GGA TGA GCA AGG CGA ACA AC | TCC TGA TTA TTG CCC CAG GC |
| *STX1B* | AGC TGC GGA GTG CGA AAG A | GTC TTC TCA TCG GGG TTG GG |
| *DNM1* | ATC GAG GGT TCT GGA GAC CA | AAG CGA GGT CAG GTG TGA AG |
| *GAPDH* | CAT CAC TGC CAC CCA GAA GAC TG | ATG CCA GTG AGC TTC CCG TTC AG |

*SLC17A6*, Solute carrier family 17 member 6; *RIMS1*, Regulating synaptic membrane exocytosis 1; *STXBP1*, Syntaxin binding protein 1; *UNC13C*, Unc-13 homolog C; *CPLX1*, Complexin 1; *CPLX2*, Complexin 2; *SNAP25*, Synaptosomal-associated protein 25; *STX1B*, Syntaxin 1B; *DNM1*, Dynamin-1; *GAPDH*, Glyceraldehyde 3-phosphate dehydrogenase.
